# Fludarabine-treosulfan versus fludarabine-melphalan or busulfan-cyclophosphamide conditioning in older AML or MDS patients – A clinical trial to registry data comparison

**DOI:** 10.1038/s41409-024-02241-2

**Published:** 2024-02-21

**Authors:** Dietrich Wilhelm Beelen, Simona Iacobelli, Linda Koster, Dirk-Jan Eikema, Anja van Biezen, Friedrich Stölzel, Fabio Ciceri, Wolfgang Bethge, Peter Dreger, Eva-Maria Wagner-Drouet, Péter Reményi, Matthias Stelljes, Miroslaw Markiewicz, Donal P. McLornan, Ibrahim Yakoub-Agha, Mohamad Mohty

**Affiliations:** 1 Department of Haematology and Stem Cell Transplantation, West German Cancer Center, University Hospital of Essen, University of Duisburg-Essen, Essen, Germany; 2 Department of Biology, University Tor Vergata of Rome, Rome, Italy; 3 EBMT Data Office Leiden, Department of Medical Statistics and Bioinformatics, Leiden University Medical Center, Leiden, Netherlands; 4 Division of Stem Cell Transplantation and Cellular Immunotherapies, Department of Internal Medicine 2, University Hospital Schleswig-Holstein, Kiel, Kiel University, Kiel, Germany; 5 Faculty of Medicine Carl Gustav Carus Dresden, Technical University Dresden, Dresden, Germany; 6 Hematology and Bone Marrow Transplantation Unit, IRCCS San Raffaele Scientific Institute, Milan, Italy; 7 Department of Hematology and Oncology, University Hospital Tübingen, Tübingen, Germany; 8 Department of Internal Medicine V, University of Heidelberg, Heidelberg, Germany; 9 Third Department of Medicine - Hematology, Internal Oncology & Pneumology, Johannes Gutenberg-University Medical Center, Mainz, Germany; 10 St. István and St. László Hospital of Budapest, Budapest, Hungary; 11 Department of Medicine A, Hematology, Oncology, Hemostaseology and Pneumology, University Hospital Münster, Münster, Germany; 12 Department of Hematology, Institute of Medical Sciences, College of Medical Sciences, University of Rzeszow, Rzeszow, Poland; 13 University College London Hospital, London, UK; 14 Centre Hospitalier Universitaire de Lille, Université Lille, INSERM U1286, Infinite, Lille, France; 15 Hospital Saint-Antoine, Sorbonne Université, Centre de Recherche Saint-Antoine (CRSA), Paris, France

**Keywords:** Clinical trials, Stem-cell therapies

## Abstract

A randomized study (acronym: MC-FludT.14/L Trial II) demonstrated that fludarabine plus treosulfan (30 g/m²) was an effective and well tolerated conditioning regimen for allogeneic hematopoietic cell transplantation (allo-HCT) in older patients with acute myeloid leukemia (AML) and myelodysplastic syndrome (MDS). To further evaluate this regimen, all 252 study patients aged 50 to 70 years were compared with similar patients, who underwent allo-HCT after fludarabine/melphalan (140 mg/m²) (FluMel) or busulfan (12.8 mg/kg)/cyclophosphamide (120 mg/kg) (BuCy) regimens and whose data was provided by the European Society for Blood and Marrow Transplantation registry. In 1:1 propensity-score matched-paired analysis (PSA) of AML patients, there was no difference in 2-year-relapse-incidence after FluTreo compared with either FluMel (*n* = 110, *p* = 0.28) or BuCy (*n* = 78, *p* = 0.98). However, 2-year-non-relapse-mortality (NRM) was lower compared with FluMel (*p* = 0.019) and BuCy (*p* < 0.001). Consequently, 2-year-overall-survival (OS) after FluTreo was higher compared with FluMel (*p* = 0.04) and BuCy (*p* < 0.001). For MDS patients, no endpoint differences between FluTreo and FluMel (*n* = 30) were evident, whereas 2-year-OS after FluTreo was higher compared with BuCy (*n* = 25, *p* = 0.01) due to lower 2-year-NRM. Multivariate sensitivity analysis confirmed all significant results of PSA. Consequently, FluTreo (30 g/m²) seems to retain efficacy compared with FluMel and BuCy, but is better tolerated by older patients.

## Introduction

Determination of the most effective and best tolerated preparative regimens for older patients with acute myeloid leukemia (AML) or myelodysplastic syndrome (MDS) remains an important research focus in the field of allogeneic hematopoietic cell transplantation (allo-HCT) [1–3]. Preparative regimens, which fuse lower organ toxicities of reduced-intensity conditioning (RIC) with potent antileukemic properties of myeloablative conditioning (MAC), are therefore provisionally termed reduced-toxicity conditioning (RTC) regimens and appear of special importance.

One promising RTC regimen is the combination of the purine analogue fludarabine and the bifunctional alkylating agent treosulfan (FluTreo), for which a particularly favorable acute organ toxicity profile in conjunction with complete and sustained donor hematopoietic chimerism after allo-HCT has been demonstrated by prospective phase II studies in adult AML and MDS patients [4–6]. In these trials, intravenous (IV) treosulfan was utilised in a total dose range between 30 and 42 grams per square meter of patient body surface area (g/m²) without any notable dose-limiting acute non-hematologic toxicities. One multicentre prospective randomized phase III study (acronym: MC-FludT.14/L Trial II, EudraCT-No: 2008–002356-18, ClinicalTrials.gov Identifier: NCT00822393; hereinafter referred to as the study) in older (i.e., 50 to 70 years of age) or comorbid AML and MDS patients compared the FluTreo regimen with a total treosulfan dose of 30 g/m² with the reference regimen of RIC fludarabine and busulfan (FluBu). This trial was designed to demonstrate at least non-inferiority of the FluTreo regimen regarding the primary composite endpoint of event-free survival within 2 years after allo-HCT with disease relapse, graft failure, or death from any cause as events [7]. The final trial analysis including all 570 randomized patients with longer follow-up met the pre-specified criteria for superiority of the FluTreo regimen [8].

To further evaluate the position of this regimen within the spectrum of the recently developed transplant conditioning intensity (TCI) scheme [9], an observational comparative analysis of all 252 study patients was performed using combinations of fludarabine (total dose: 150 mg/m² IV) and melphalan (total dose: 140 mg/m² IV) (FluMel), or busulfan (total dose: 12.8 mg/kg IV) and cyclophosphamide (total dose: 120 mg/kg) (BuCy) as comparator regimens.

The FluMel regimen is assigned to the intermediate TCI category and was selected for this comparison because observational single-center and HCT-registry studies suggested lower relapse risks without compromising non-relapse mortality (NRM) in older AML and MDS patients compared with the RIC-FluBu reference regimen of the MC-FludT.14/L Trial II study [3, 10–13]. Although superior antileukemic properties of FluMel have not been substantiated by randomized studies to date, this putative favorable effect unequivocally contributed to its widely application during recent years. In considering that the MC-FludT.14/L Trial II study demonstrated significantly lower NRM of the FluTreo regimen opposed to the RIC-FluBu regimen, a comparison of FluTreo to the FluMel regimen in older AML/MDS patients appeared obvious and appropriate. The intermediate TCI busulfan (12.8 mg/kg IV) and fludarabine regimen was not considered for this comparison, because this regimen failed to demonstrate any durable beneficial effect on relapse risk, leukemia-free and overall survival for AML patients in 1st or 2nd complete remission (CR) in the prospective randomized comparison to the BuCy regimen [14]. Therefore and because it is the only pharmacologic conditioning regimen assigned to the high TCI category, the previously widely applied BuCy regimen was selected for comparison to FluTreo. This comparison thus aimed to contrast major outcomes of the only pharmacologic high TCI category regimen to the MC-FludT.14/L Trial II study regimen. Since the FluTreo regimen as applied in the MC-FludT.14/L Trial II study has so far not been evaluated in comparison to BuCy, this comparison should provide further insights regarding its safety and efficacy in older AML and MDS patients.

Eligible patients who received either FluMel or BuCy conditioning for allo-HCT and were documented within the European Society for Blood and Marrow Transplantation (EBMT) registry served as real-world comparators for the study patients. Propensity score (PS)-based matched-paired univariate analysis (PSA) and multivariable Cox proportional-hazards regression analysis for sensitivity testing were performed to compare estimates of overall survival (OS), cumulative relapse incidence (RI), and NRM within 2 years after allo-HCT between FluTreo and FluMel or BuCy regimens.

## Methods

### Study design

This was a retrospective EBMT registry-based study contracted by the sponsor of the pivotal MC-FludT.14/L Trial II study (medac GmbH, 22880 Wedel, Germany) after approval of the statistical analysis plan by the EBMT Acute Leukaemia and Chronic Malignancies Working Parties of the EBMT institutional review board and conducted in accordance with the Declaration of Helsinki and Good Clinical Practice guidelines. Selection of registry patients complied with eligibility criteria of study patients as previously published and summarized in Table 1 [7]. The recruitment period of all 252 study patients between 50 to 70 years of age (median age: 61 years) ranged between June 2013 and December 2016 (median: 2015). The follow-up period of study patients was terminated at the end of December 2017. To ensure sufficient sample sizes, registry patients were included from 2010 to 2016 (median: 2013). However, due to its retrospective nature, sample sizes of this study were not chosen to ensure adequate power for detection of a pre-specified effect size.The FluMel regimen was restricted to fludarabine 150 mg/m² IV and melphalan 140 mg/m² IV. The BuCy regimen consisted of busulfan 12.8 mg/kg IV and cyclophosphamide 120 mg/kg IV. As baseline prophylaxis of graft-versus-host disease (GvHD) short-course methotrexate and ciclosporin was applied in the study, but this was not considered in comparisons with registry patients. However, as for study patients, anti-thymocyte globulin (ATG) prophylaxis was mandatory for allo-HCT using matched unrelated donors (MUD) and was precluded for matched sibling donors (MSD) in registry patients. Further, no graft manipulation technique for GvHD prophylaxis was permitted [7].

**Table 1:** Eligibility criteria for comparison of EBMT registry to MC-FludT.14/L Trial II study patients.

1. Patient age between 50 and 70 years
2. Karnofsky performance score of at least 60
3. Primary or secondary AML in complete remission or MDS^†^
4. Matched (i.e., HLA-identical) sibling donor or matched unrelated donor^‡^
5. First allogeneic hematopoietic cell transplantation
6. Peripheral blood or bone marrow stem cell graft source

*AML* Acute myeloid leukemia, *MDS* Myelodysplastic syndrome, *HLA* human leucocyte antigen.

^†^Regardless of WHO subtype or IPSS-R risk score.

^‡^Matched at least at HLA-loci A, B, C, DRB1, DQB1.

All data of study patients was provided as analysis data model subject level datasets in accordance with the Clinical Data Interchange Standards Consortium to the EBMT registry data office, Leiden, The Netherlands. Signed informed consent for pseudonymized scientific analysis of study data had been obtained from all study patients as part of the MC-FludT.14/L study protocol. All registry patient data was derived from MED-A documentation, which retrieve essential individual information on patient, donor, and pretransplant disease characteristics as well as on transplant procedures and outcome measures from EBMT member institutions. All registry patients gave signed informed consent for data submission and scientific analysis within the EBMT registry.

### Statistical analysis

Outcome variables were defined in accordance with internal consensus guidelines [15]. For comparison of baseline patient, disease, and treatment characteristics continuous data between study and registry patients Mann–Whitney’s test was used and baseline categorical data were compared using the Pearson’s chi-squared test.

Two approaches were selected for comparisons of clinical outcome endpoints between study and registry patients. First, PSA was used to reduce confounding due to differences between study and registry patients. The PS was calculated using binary logistic regression models [16, 17]. Matching was separately performed for both disease category and comparator registry conditioning regimen, thus resulting in 4 distinct comparator groups (Supplementary Table S1). The matching ratio was 1:1 in order to prioritise reduction of confounding factors over precision of effect estimates. The nearest neighbor matching approach was generally applied with selection of pairs within a standard caliper of 0.2 standard deviations of the respective PS [18]. The following independent pre-transplantation variables were included in models for PS calculation: patient age and sex, stratified disease stage (AML: 1st CR [CR1] versus CR > 1 according to revised WHO classification 2008 [19]; MDS: bone marrow blast content up to 10% versus more than 10% to 20%), disease origin (primary versus secondary, i.e., therapy-related), stratified disease risk group [AML: favorable versus intermediate versus adverse risk based on the European LeukemiaNet standardized reporting for correlation of cytogenetic and molecular genetic data with clinical data 2010 (CR > 1 stratified to the adverse risk group); MDS: (very) low versus intermediate versus (very) high according to the Revised International Prognostic Scoring System (IPSS-R) 2012] [20, 21], stratified hematopoietic cell transplantation comorbidity index (HCT-CI) [22], and stratified Karnofsky performance score (KPS). The following transplant-related variables were included: donor type (MSD versus MUD), donor age, female donor to male recipient versus other gender combinations, donor and patient cytomegalovirus (CMV) serostatus combinations, graft source (bone marrow [BM] versus peripheral blood [PB]), and (whenever possible) year of HCT.

The similarity of matched groups was assessed by descriptive statistics and significance testing (paired *t*-test for continuous variables and paired McNemar test for categorical variables). Estimates of OS and 95% confidence intervals (±95% CI) were calculated by the product-limit method and heterogeneity of survival distributions was tested using the unpaired and paired log-rank test [23]. RI and NRM estimates (±95% CI) were calculated as cumulative incidences with mutually competing events [24]. Heterogeneity of cumulative incidence functions was tested by Gray’s method [25].

As an alternative approach to control confounding, multivariable regression models were applied using all registry patients fulfilling the eligibility criteria and all 252 study patients. Cox proportional-hazards model was used for both OS hazard and for RI and NRM cause-specific hazards. Differences between treatment groups were evaluated as hazard ratios (HR) (with 95% CI) and the corresponding p-values were derived from the unadjusted Wald test [26]. All mentioned variables were only considered as adjustment factors for outcome and respective HR estimation of the main treatment effect. All p-values shown refer to two-sided tests. Due to its explorative character, no adjustments for multiple testing were performed in this study.

To account for differences in follow-up periods of study (median follow-up: 30 months) and registry (median follow-up: 38 months) patients, outcomes were censored at 2 years after HCT for all comparisons. Patients without events within 2 years were censored at last follow-up dates.

## Results

### Patient, disease, and transplant characteristics

A total of 968 registry patients were identified, who met the eligibility criteria and for whom both comparator regimens were documented without any additional cytotoxic agents (Supplementary Table S1).

In AML patients, comparison between FluTreo study (*n* = 174) and FluMel registry (*n* = 256) patients resulted in younger donor age, higher proportions of HCT-CI > 2, KPS < 90, primary disease origin, and MUD-HCT in study patients (Table 2). Comparison to BuCy registry patients (*n* = 503) revealed higher patient age, younger donor age, higher proportions of HCT-CI > 2, KPS < 90, intermediate/adverse disease risk, MUD-HCT, negative donor and patient CMV serostatus, and PB grafts, the last two probably related to the higher proportion of MUD-HCT in study patients (Table 2).

**Table 2:** Acute myeloid leukemia patient and disease characteristics.

	AML Regimens							
	FluMel		*p* value	BuCy		*p* value	FluTreo	
Number of patients	256			503			174	
Patient age (years)			0.78			<0.001		
Median (min-max)	60.9 (50.1-69.9)			54.4 (50.0-66.8)			61.0 (50.0-70.0)	
[IQR]	[56.3-64.0]			[52.3-57.3]			[55.0-65.0]	
Donor age (years)			0.02			<0.001		
Median (min-max)	40.6 (20.2-71.1)			45.7 (18.5-72.7)			35.0 (18.0-74.0)	
[IQR]	[27.6-56.7]			[31.7-53.9]			[25.0-50.0]	
Interval from D_x_ to HCT (months)			0.06			0.11		
Median (min-max)	4.9 (1.2-69.0)			5.7 (0.8-177.5)			5.2 (1.7-46.9)	
[IQR]	[3.4-7.6]			[4.2-9.1]			[3.8-9.3]	
	***N***	**%**	***p*** **value**	**N**	**%**	***p*** **value**	***N***	**%**
Patient sex			0.08			0.30		
Male	125	48.8		266	52.9		100	57.5
Female	131	51.2		237	47.1		74	42.5
HCT-CI			<0.001			<0.001		
≤2	112	43.8		202	40.2		79	45.4
>2	28	10.9		38	7.5		95	54.6
Missing	116	45.3		263	52.3			
Karnofsky performance score			<0.001			<0.001		
<90	40	15.6		77	15.3		71	40.8
≥90	193	75.4		408	81.1		92	52.9
Missing	23	8.9		18	3.6		11	6.3

	**AML Regimens**							
	**FluMel**			**BuCy**			**FluTreo**	
	***N***	**%**	***p*** **value**	***N***	**%**	***p*** **value**	***N***	**%**
Disease of secondary origin			0.027			0.27		
No	229	89.5		468	93.0		166	95.4
Yes	27	10.5		35	7.0		8	4.6
Disease stage			0.43			0.34		
CR1	212	82.8		415	82.5		149	85.6
CR>1	44	17.2		88	17.5		25	14.4
Disease risk			0.325			0.003		
Favorable	13	5.1		40	7.9		13	7.5
Intermediate	48	18.8		59	11.7		65	37.4
Adverse	54	21.1		112	22.3		96	55.2
Missing	141	55.1		292	58.1		0	0.0
Donor type			0.020			<0.001		
MSD	87	34.0		265	52.7		41	23.6
MUD	169	66.0		238	47.3		133	76.4
Donor-patient gender combinations			0.284			0.747		
F to M	43	16.8		100	19.9		37	21.3
Other combinations	208	81.3		397	78.9		137	78.7
Missing	5	0.2		6	1.2		0	0.0
Donor-patient CMV serostatus			0.611			0.006		
neg-neg	67	26.2		64	12.7		41	23.6
neg-pos	60	23.4		123	24.5		51	29.3
pos-neg	21	8.2		34	6.8		12	6.9
pos-pos	100	39.1		250	49.7		70	40.2
missing	8	3.1		32	6.3		0	0.0
Graft source			0.147			<0.001		
BM	19	7.4		72	14.3		7	4.0
PB	237	92.6		431	85.7		167	96.0

*AML* acute myeloid leukemia, *FluMel* fludarabine/melphalan, *BuCy* busulfan/cyclophosphamide, *FluTreo* fludarabine/treosulfan, *D*_*x*_ diagnosis, *HCT* hematopoietc cell transplantation, *CI* comorbidity index, *CR* complete remission, *MSD* matched sibling donor, *MUD* matched unrelated donor, *F to M* female donor to male patient, *CMV* cytomegalovirus, *BM* bone marrow, *PB* peripheral blood, *IQR* interquartile range, *min* minimum, *max* maximum, *neg* negative, *pos* positive.

*p* values for comparisons of continuous variables of FluMel or BuCy to FluTreo were calculated using the Mann–Whitney test. *p* values for comparisons of categorical variables were calculated by Pearson’s Chi Squared test.

Similar patterns of significant differences between FluTreo (*n* = 78) and FluMel (*n* = 82) or BuCy (*n* = 127) groups were notable for MDS patients especially with regard to younger donor age, higher proportion of HCT-CI > 2, KPS < 90, and MUD-HCT (Table 3). Overall, FluTreo patients in particular had higher comorbidity burden, lower performance status and underwent MUD-HCT more frequently as opposed to EBMT registry patients of both comparator regimens.

**Table 3:** Myelodysplastic syndrome patient and disease characteristics.

	MDS Regimens							
	FluMel		*p* value	BuCy		*p* value	FluTreo	
Number of patients	82			127			78	
Patient age (years)			0.30			<0.001		
Median (min-max)	62.2 (50.1–69.9)			55.9 (50.0–67.2)			61.0 (50.0–70.0)	
[IQR]	[56.6–64.9]			[53.0–57.8]			[56.0–65.0]	
Donor age (years)			0.002			0.01		
Median (min-max)	49.3 (19.2–73.7)			43.8 (21.2–74.5)			33.5 (18.0–65.0)	
[IQR]	[31.5–59.4]			[30.8–52.2]			[27.0–46.0]	
Interval from D_x_ to HCT (months)			0.13			0.06		
Median (min-max)	8.3 (0.9–167.7)			8.4 (2.0–110.8)			6.4 (0.5–135.9)	
[IQR]	[4.9–18.2]			[5.0–16.9]			[4.1–17.0]	
	***N***	**%**	***p*** **value**	***N***	**%**	***p*** **value**	***N***	**%**
Patient sex			0.47			0.037		
Male	62	75.6		71	55.9		55	70.5
Female	20	24.4		56	44.1		23	29.5
HCT-CI			<0.001			<0.001		
≤2	40	48.8		47	37.0		33	42.3
>2	6	7.3		8	6.3		45	57.7
Missing	36	43.9		72	56.7		0	0.0
Karnofsky performance score			<0.001			<0.001		
<90	21	25.6		26	20.4		47	60.3
≥90	50	61.0		97	76.4		29	37.2
Missing	11	13.4		4	3.2		2	2.5

	**MDS Regimens**							
	**FluMel**			**BuCy**			**FluTreo**	
	***N***	**%**	***p*** **value**	***N***	**%**	***p*** **value**	***N***	**%**
Disease of secondary origin			0.81			0.29		
No	59	72.0		99	78.0		61	78.2
Yes	16	19.5		16	12.6		15	19.2
Missing	7	8.5		12	9.4		2	2.6
Disease stage			0.014			0.538		
BM blasts <10%	41	50.0		45	35.4		25	32.1
BM blasts 10–20%	39	47.6		79	62.2		53	67.9
Missing	2	2.4		3	2.4		0	0.0
Pretransplant treatment			0.15			0.022		
Untreated	30	36.6		40	31.5		38	48.7
Treated	50	61.0		83	65.3		40	51.3
Missing	2	2.4		4	3.2		0	0.0
Disease risk score			0.60			0.763		
(very) low	16	19.5		22	17.3		32	41.0
Intermediate	5	6.1		13	10.2		18	23.1
(very) high	9	11.0		9	7.1		18	23.1
Missing	52	63.4		83	65.4		10	12.8
Donor type			<0.001			<0.001		
MSD	38	46.3		61	48.0		14	17.9
MUD	44	53.7		66	52.0		64	82.1
Donor-patient gender combinations			0.043			0.85		
F to M	21	25.6		19	15.0		11	14.1
Other combinations	56	68.3		107	84.2		67	85.9
Missing	5	6.1		1	0.8		0	0.0
Donor-patient CMV serostatus			0.74			0.20		
neg-neg	26	31.7		34	26.8		28	35.9
pos-neg	9	11.0		10	7.9		12	15.4
neg-pos	17	20.7		30	23.6		13	16.7
pos-pos	29	35.4		43	33.9		25	32.1
Missing	1	1.2		10	7.9		0	0.0
Graft source			0.59			0.09		
BM	2	2.4		8	6.3		1	1.3
PB	80	97.6		119	93.7		77	98.7

*MDS* myelodysplastic syndrome, *FluMel* fludarabine/melphalan, *BuCy* busulfan/cyclophosphamide, *FluTreo* fludarabine/treosulfan, *D*_*x*_ diagnosis, *HCT* hematopoietc cell transplantation, *CI* comorbidity index, BM bone marrow, *MSD* matched sibling donor, *MUD* matched unrelated donor, *F to M* female donor to male patients, *CMV* cytomegalovirus, *PB* peripheral blood, *IQR* interquartile range, *min* minimum, *max* maximum, *neg* negative, *pos* positive.

*p* values for comparisons of continuous variables of FluMel or BuCy to FluTreo were calculated using the Mann–Whitney test. *p* values for comparisons

of categorical variables were calculated using the Pearson’s Chi Squared test.

### Propensity score matched-paired analysis

For 1:1 PSA outcome comparisons between FluTreo and FluMel regimens in AML patients, 110 sufficiently matched pairs were identified, representing 63% of FluTreo and 43% of eligible FluMel patients. Corresponding figures of comparisons to the BuCy regimen were 78 matched pairs, representing 45% of FluTreo and 16% of eligible BuCy patients (Supplementary Table S2). The single remaining significant difference on PSA was a higher proportion of HCT-CI ≤ 2 in FluMel patients (*p* < 0.001) (Supplemetary Table S2).

For comparison between FluTreo and FluMel regimens in MDS patients, 30 matched pairs were identified, representing 38% and 37% of eligible patients, respectively. Corresponding figures for comparison between FluTreo and BuCy regimens were 25 matched pairs, representing 32% of FluTreo and 20% of BuCy patients (Supplementary Table S3). The only significant difference between FluTreo and FluMel or BuCy patients was a substantially higher proportion of HCT-CI ≤ 2 in both comparator regimen groups (FluMel *p* = 0.005; BuCy *p* < 0.001) (Supplementary Table S3). Notably, these differences were not reflected by stratified KPS, which was almost equally distributed in both comparisons.

**Fig. 1:**
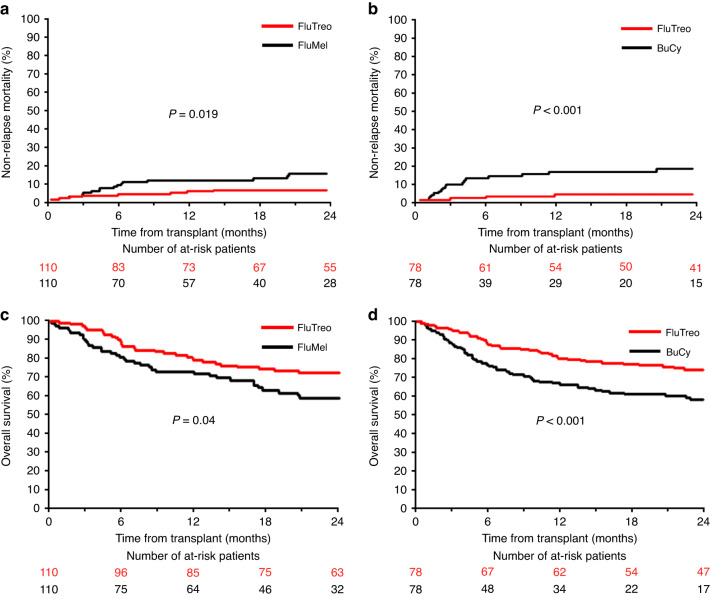
Outcome comparison of FluTreo with FluMel or BuCy by propensity score 1:1 matched-pairs analysis of acute myeloid leukemia patients. Comparison of cumulative incidence of non-relapse mortality between (**a**) FluTreo and FluMel (**b**) FluTreo and BuCy; comparison of overall survival between (**c**) FluTreo and FluMel (**d**) FluTreo and BuCy; non-relapse mortality curves represent cumulative incidence estimates with relapse as competing risk, overall survival curves represent product-limit estimates; p-values for comparisons of non-relapse mortality were calculated by Gray’s test, p-values for comparisons of overall survival were calculated by log-rank test; FluTreo Fludarabine/Treosulfan with a total dose of 30 g/m², FluMel Fludarabine/ Melphalan with a total dose of 140 mg/m²; BuCy Busulfan with a total dose of 12.8 mg/kg and Cyclophosphamide with a total dose of 120 mg/kg (all agents given intravenously).

For AML patients, comparison of FluTreo with FluMel or BuCy regimens resulted in similar 2-year RI, which were in the range between 25% and 31% (Table 4). In contrast, the 2-year NRM of FluTreo was substantially lower compared with FluMel and BuCy patients (Table 4, Fig. 1a, b). The difference in 2-year NRM between FluTreo and FluMel regimens was significant only in unpaired comparison (*p* = 0.019) (Table 4, Fig. 1a), but nevertheless appears meaningful considering the significantly higher proportion of HCT-CI > 2 as an unfavorable influential factor of NRM for FluTreo patients (Suppl. Table S2). The lower 2-year NRM of FluTreo patients translated into higher 2-year OS compared with FluMel and BuCy patients (Table 4, Fig. 1c, d). In accordance with the difference in 2-year NRM between FluTreo and FluMel regimens, the difference of 2-year OS between both regimens was significant only in unpaired comparison (*p* = 0.04) (Table 4). Between FluTreo and BuCy regimens, however, the 2-year OS was significantly different in paired (*p* < 0.001) as in unpaired (*p* < 0.001) comparison (Table 4).

**Table 4:** Propensity score 1:1 matched-paired analysis of clinical endpoints at 2 years after allogeneic HCT.

	Acute myeloid leukemia					
	Relapse	*p* value	Non-relapse mortality	*p* value	Overall survival	*p* value
FluMel (*n* = 110)	24.7% (15.8–33.6)	0.28 (0.11)	17.5% (9.6–25.5)	0.019 (0.11)	58.7% (48.3–69.1)	0.04 (0.21)
FluTreo (*n* = 110)	30.6% (21.9–39.4)		6.4% (1.8–11.0)		72.7% (63.7–80.7)	
BuCy (*n* = 78)	30.3% (18.6–42.0)	0.98 (0.46)	23.5% (13.1–33.9)	<0.001 (0.001)	49.2% (36.4–62.1)	<0.001 (<0.001)
FluTreo (*n* = 78)	29.1% (18.8–39.4)		3.9% (0.0–8.2)		76.4% (66.8–85.9)	

	Myelodysplastic syndrome					
	Relapse	*p* value	Non-relapse mortality	*p* value	Overall survival	*p* value
FluMel (*n* = 30)	23.8% (5.1–42.5)	0.50 (0.74)	12.5% (0.0–25.9)	0.72 (0.71)	56.5% (33.9–79.1)	0.57 (0.62)
FluTreo (*n* = 30)	13.3% (1.2–25.5)		16.7% (3.3–30.0)		70.0% (53.6–86.4)	
BuCy (*n* = 25)	25.8% (1.8–49.9)	0.098 (0.32)	43.1% (17.2–69.0)	0.18 (0.13)	30.5% (6.1–54.9)	0.01 (0.01)
FluTreo (*n* = 25)	4.0% (0.0–11.7)		24.0% (7.3–40.7)		72.0% (54.4–89.6)	

*FluMel* fludarabine/melphalan, *BuCy* busulfan/cyclophosphamide, *FluTreo* fludarabine/treosulfan.

*p* values for comparisons of relapse incidence and non-relapse mortality were calculated using Gray’s test, *p* values for comparisons of overall survival were calculated using the log-rank test, all *p* values in parentheses were calculated using the log-rank test stratified on each matched pair.

The only significant difference in the PSA outcome comparisons in MDS patients was a higher 2-year OS of FluTreo compared with BuCy patients (72% vs 31%; *p* = 0.01) (Table 4). This was mostly (albeit not significantly) attributable to lower 2-year RI and NRM of FluTreo patients. Notably, in view of median patient ages being above 60 years, 2-year NRM of FluTreo and FluMel patients appeared comparably favorable. As expected for older MDS patients, the BuCy regimen was associated with a particularly high 2-year NRM, but without any discernible benefit regarding 2-year RI (Table 4).

### Multivariate comparison of outcomes

Multivariate outcome analysis included all eligible registry and study patients and those variables displayed in Tables 2 and 3.

For AML patients, comparison of FluTreo with FluMel or BuCy regimens completely corroborated all significant results obtained by PSA for 2-year NRM and OS endpoints (Table 5, Fig. S1). Accordingly, no difference of 2-year RI between FluTreo and both comparator regimens was observed by sensitivity testing (Table 5).

**Table 5:** Multivariate Cox regression sensitivity analysis of clinical endpoints at 2 years after allogeneic HCT.

	Acute myeloid leukemia					
	Relapse		Non-relapse mortality		Overall survival	
	HR (95% CI)	*p* value	HR (95% CI)	*p* value	HR (95% CI)	*p* value
FluMel (*n* = 256) *vs* FluTreo (*n* = 174)	1 0.84 (0.45 – 1.57)	0.588	1 0.26 (0.12 – 0.56)	0.001	1 0.34 (0.2 – 0.57)	<0.001
BuCy (*n* = 503) *vs* FluTreo (*n* = 174)	1 0.67 (0.40 – 1.12)	0.127	1 0.31 (0.15 – 0.66)	0.002	1 0.48 (0.3 – 0.78)	0.003

	Myelodysplastic syndrome					
	Relapse		Non-relapse mortality		Overall survival	
	HR (95% CI)	*p* value	HR (95% CI)	*p* value	HR (95% CI)	*p* value
FluMel (*n* = 82) *vs* FluTreo (*n* = 78)	n. a.	0.57	1 0.49 (0.24 – 1.02)	0.057	1 0.6 (0.32 – 1.14)	0.117
BuCy (*n* = 127) *vs* FluTreo (*n* = 78)	n. a.	0.33	1 0.46 (0.19 – 1.11)	0.085	1 0.29 (0.14 – 0.60)	0.001

*FluMel* fludarabine/melphalan, *FluTreo* fludarabine/treosulfan, *BuCy* busulfan/cyclophosphamide.

*p* values and hazard ratios (95% CI) were derived from Cox regression analysis (overall survival) and cause-specific Cox regression analysis (relapse and non-relapse mortality) after adjustment for patient and disease characteristics, *n. a*. not applicable because the required number of events for HR calculation as pre-specified by the statistical analysis plan (*n* = 50) was not attained.

For MDS patients, results of 2-year NRM and OS obtained by PSA were likewise corroborated in that only 2-year OS between FluTreo and BuCy regimens was significantly different (Tables 4 and 5).

## Discussion

The provisionally termed RTC regimens pursue the therapeutic goal of improving outcome of allo-HCT by fusing lower non-hematologic organ toxicities of RIC with the higher antileukemic efficacy of MAC regimens. This is especially important for older AML and MDS transplant candidates, for whom MAC regimens are associated with unacceptably high NRM, but RIC regimens may compromise outcomes due to increased relapse rates as demonstrated by a single [27], but not all randomized trials [14, 28, 29].

In contrast to phase II studies or retrospective single-center and registry analyses, which predominantly evaluated FluTreo regimens with total treosulfan doses of 36 and 42 g/m² [30–37], the MC-FludT.14/L Trial II study was performed with a total treosulfan dose of 30 g/m² after it became apparent that the originally applied total dose of 42 g/m² led to substantially prolonged neutropenia compared with the RIC-FluBu reference regimen [7]. The recently proposed intensity weighted TCI risk scheme categorized this FluTreo regimen as a low intensity regimen with a score of only 1.5 [9].

For AML patients, the present results suggest that the FluTreo regimen as applied in the MC-FludT.14/L Trial II study is associated with significantly lower NRM compared with the intermediate intensity FluMel regimen. In contrast, one recent EBMT registry study comparing a total dose of 42 g/m² treosulfan to 140 mg/m² melphalan in otherwise similar AML patients, revealed only slightly lower NRM after FluTreo, which, however, appears substantially higher than in the present analysis [35]. Furthermore, this study revealed significantly lower RI after FluMel, which was not notable in the present analysis. The substantially lower NRM together with comparable RI translated into significantly higher OS of FluTreo patients on multivariate analysis in the present analysis. In contrast, the lower RI obtained in FluMel patients of the EBMT registry study did not result in higher OS because it was counterbalanced by NRM [35]. Whether these differences actually reflect effects of treosulfan dose intensity cannot be clarified currently, but the present results at least argue against substantially stronger antileukemic properties of FluMel compared with the FluTreo regimen, even at a total treosulfan dose of 30 g/m².

Direct comparisons of FluTreo to the BuCy regimen are hardly ever available for older AML patients in hematologic CR, because this regimen is generally considered not suitable due to its well-documented higher NRM in comparison to MAC- and RIC-FluBu regimens [14, 27]. Nonetheless, it appears pertinent to contrast major outcomes of the FluTreo regimen as applied in the MC-FludT.14/L Trial II study to an established pharmacologic regimen assigned to the high TCI category in order to elucidate the position of this FluTreo regimen within the spectrum of conditioning intensities [9]. In PSA as well as in multivariate analysis, the 2-year RI was completely congruent after both regimens, but 2-year NRM was nearly 3-fold lower and consequently, 2-year OS was significantly higher for FluTreo patients. Thus, similar to its comparison with the FluMel regimen, the present results likewise support that the antileukemic efficacy of this FluTreo regimen is not inferior compared with the BuCy regimen but reduces NRM substantially and thereby improves OS of older AML patients.

Comparisons between the FluTreo and the FluMel regimen in MDS patients did not reveal any perceivable outcome differences in the present analysis. The Center for International Blood and Marrow Transplant Research recently reported results of comparisons between FluMel and RIC-FluBu regimens in older MDS patients which indicated that the FluMel regimen is associated with lower 3-year RI and higher 3-year OS [13]. Taken together, these results speak for prospective comparisons between FluMel and FluTreo regimens in older MDS patients. Our comparisons with BuCy patients likewise did not reveal any evidence for an increased 2-year RI after the FluTreo regimen. Instead, both 2-year RI and NRM tended to be lower, and consequently, 2-year OS after the FluTreo regimen was even significantly higher. These results are in accordance with recently reported comparative results between FluTreo regimens comprising different treosulfan dosages and categorized RIC or MAC regimens in MDS patients, which also indicated that RI is not increased after FluTreo as opposed to MAC regimens [34]. As in the present analysis, FluTreo regimens were also associated with superior OS compared with MAC regimens due to lower NRM [34].

As with any retrospective analysis, the present study has inevitable limitations, which raise caveats on interpretation of obtained results. This particularly applies to potential selection biases caused by heterogeneous distribution of established and, even more important, of undetermined influencial factors which can only be properly balanced by randomization. The issue of selection bias was considered as much as possible by using 1:1 PSA, which took the most important patient and clinical disease characteristics for the major outcome endpoints during selection of control patients into account. The rigorous eligibility criteria for this selection and, most prominently, restriction to older patient age, substantially diminished eligible control patient numbers from the registry. An insurmountable limitation for the identification of completely matched pairs was the substantially higher prevalence of pretransplant comorbidities in study patients, which resulted in imbalanced distributions of the HCT-CI > 2 category compared with control patients. This reflects the fact that the MC-FludT.14/L Trial II study protocol was particularly designed for older and comorbid patients considered not suitable for conditioning regimens with higher conditioning intensity. Further, information on pretransplant genetic disease risk factors was available only for those control patients included in PSA [20, 21]. Thus, pretransplant genetic disease risk stratification, which was established and commonly used during the time period, in which the MC-FludT.14/L Trial II study was conducted, could not be accounted for in the multivariable sensivity analysis. Moreover, assessment of measurable residual disease (MRD) for disease risk stratification at the time of transplant could not be implemented in the MC-FludT.14/L Trial II study protocol, because standardized and validated methods as well as recommendations for routine MRD evaluation were not yet established for molecular disease-specific alterations of AML and were only in early developmental stages for MDS during study design and conduct. Thus, commonly accepted and widely applicable MRD evaluation was not yet available for disease risk stratification in the present study. The potentially added value of MRD evaluation at the time of transplant for disease risk stratification remains, however, questionable for this study, because the comparisons of FluTreo with FluMel and BuCy regimens revealed no evidence of differing relapse risks between study and control patients. Nonetheless, pretransplant MRD evaluation might have unmasked potential differences of an otherwise undetectable disease burden between study and control patients, who were in complete remission pretransplant. That, however, seems unlikely in consideration of relatively uniform pretransplant treatment algorithms in the eligible patient population. In terms of the comparisons of relapse incidences, the MC-FludT.14/L Trial II study protocol assessed all posttransplant interventions such as donor lymphocyte infusions, hypomethylating agents, and other disease-directed therapeutic approaches after allo-HCT as relapse events [7]. These interventions could not be accounted for in control patients due to missing information. Thus, this could also represent an observational bias, which might even imply overestimation of the RI in study patients. Nonetheless, our comparisons between study and real-world registry patients provide some important clues on the conditioning intensity of the FluTreo regimen as applied in the MC-FludT.14/L Trial II study and on future conceptions for randomized studies in older AML and MDS patients.

In summary, comparison between this FluTreo regimen and the intermediate conditioning intensity FluMel regimen support similar antileukemic efficacy, but better tolerability of the FluTreo regimen in older AML patients in CR. Both regimens led to equivalent outcomes in older MDS patients. Compared with the high conditioning intensity BuCy regimen, the FluTreo regimen was associated with substantially lower NRM, which translated into better OS in older AML and MDS patients. Together, these results suggest that the FluTreo regimen as applied in the MC-FludT.14/L Trial II study can be categorized as an RTC regimen with similar efficacy as regimens with intermediate or even high conditioning intensity in older AML and MDS patients.

### Supplementary information

Supplemental Material

## Data Availability

The MC-FludT.14/L Trial II study research data at patient level are not shared.
