# Supplementary material for: Fludarabine-treosulfan versus fludarabine-melphalan or busulfan-cyclophosphamide conditioning in older AML or MDS patients – A clinical trial to registry data comparison
Source: Bone Marrow Transplant. 2024 Feb 21;59(5):670–9. doi: 10.1038/s41409-024-02241-2 (PMC11073976; doi:10.1038/s41409-024-02241-2)
Supplement: Supplementary file 1 — Supplemental Material [file 41409_2024_2241_MOESM1_ESM.pdf]

SUPPLEMENTAL MATERIAL

Supplementary Table S1: Number of patients by treatment group and disease category

		Control Cohort† (N=968)		Treatment Cohort‡ (N=252)
		**FluMel (EBMT)**	**BuCy (EBMT)**	**FluTreo (CT)**
**Disease**	**MDS**	82	127	78
	**AML**	256	503	174
**Total**		338	630	252

AML denotes acute myeloid leukaemia; MDS denotes myelodysplastic syndrome

† FluMel: fludarabine 150 mg/m² IV over 5 days and melphalan 140 mg/m² IV over 1 or 2 day(s); BuCy: busulfan 12.8 mg/kg over 4 days and cyclophosphamide 120 mg/kg over 2 days

‡ FluTreo: fludarabine 150 mg/m² IV over 5 days and treosulfan 30 g/m² IV over 3 days

Supplementary Table S2: Patient and disease characteristics of matched pairs of acute myeloid leukemia patients.

	FluMel	FluTreo		BuCy	FluTreo	
**Number of patients**	110	110	**p value**	78	78	**p value**
**Patient age (years)**			0.50			0.51
**Median (min-max)**	61.4 (50.0-69.6)	61.0 (50.0-70.0)		57.2 (50.2-66.8)	56.0 (50.0-70.0)	
**[IQR]**	[57.2-63.9]	[54.3-65.0]		[53.7-61.2]	[53.0-61.0]	
**Donor age (years)**			0.25			0.57
**Median (min-max)**	32.6 (20.2-68.4)	38.5 (18.0-74.0)		35.1 (18.5-66.0)	38.0 (18.0-68.0)	
**[IQR]**	[24.8-45.4]	[26.3-50.0]		[28.5-49.4]	[26.0-53.0]	
**Interval from D_x to HCT (months)**			0.78			0.26
**Median (min-max)**	4.9 (1.6-69.0)	5.4 (2.0-45.9)		5.2 (1.6-55.7)	5.9 (1.7-45.8)	
**[IQR]**	[3.4-8.0]	[3.6-9.2]		[3.9-7.3]	[4.1-9.4]	
**Patient sex (%)**			0.49			>0.99
**- male**	57.3	52.7		62.8	62.8	
**- female**	42.7	47.3		37.2	37.2	
**HCT-CI (%)**			<0.001			0.64
**- ≤ 2**	72.4	40.9		66.0	65.4	
**- > 2**	27.6	59.1		34.0	34.6	
**Karnofsky performance score (%)**			0.35			0.24
**- < 90**	29.0	33.3		36.1	22.2	
**- ≥ 90**	71.0	66.7		63.9	77.8	
**Disease of secondary origin (%)**			0.74			0.71
**- No**	96.4	95.5		94.9	96.2	
**- Yes**	3.6	4.5		5.1	3.8	

Supplementary Table S2 continued:

	FluMel	FluTreo		BuCy	FluTreo	
			**p value**			**p value**
**Disease stage (%)**			0.86			0.62
**- CR1**	83.6	84.5		87.2	84.6	
**- CR>1**	16.4	15.5		12.8	15.4	
**Disease risk (%)**			0.05			<0.001
**- favorable**	11.8	6.4		29.4	6.4	
**- intermediate**	40.9	37.3		29.4	39.7	
**- adverse**	47.3	56.4		41.2	53.8	
**Donor type (%)**			0.46			0.74
**- MSD**	21.8	26.4		26.9	29.5	
**- MUD**	78.2	73.6		73.1	70.5	
**Donor-patient gender combinations (%)**			0.85			0.58
**- F to M**	18.3	18.2		20.8	24.4	
**- other combinations**	81.7	81.8		79.2	75.6	
**Donor-patient CMV serostatus (%)**			0.96			0.98
**- neg-neg**	22.2	24.8		22.0	19.2	
**- pos-neg**	8.3	7.3		9.1	9.0	
**- neg-pos**	27.8	26.4		24.7	25.6	
**- pos-pos**	41.7	41.8		44.2	46.2	
**Graft source (%)**			>0.99			>0.99
**- BM**	4.5	4.5		3.8	3.8	
**- PB**	95.5	95.5		96.2	96.2	

FluMel fludarabine/melphalan, *BuCy* busulfan/cyclophosphamide, FluTreo treosulfan, D_x diagnosis, *HCT* haematopoietc cell transplantation, *CI* comorbidity index, *CR* complete remission, *MSD* matched sibling donor, *MUD* matched unrelated donor, *F to M* female donors for male patients, *CMV* cytomegalovirus HCT, *BM* bone marrow, *PB* peripheral blood

P-values for comparisons of continuous variables of FluMel or BuCy to FluTreo were calculated by paired T-test; p-values for comparisons of categorical variables were calculated by paired McNemar's test or by Pearson's Chi-squared test for 2 x >2 tables.

Supplementary Table S3: Patient and disease characteristics of matched pairs of myelodysplastic syndrome patients

	FluMel	FluTreo		BuCy	FluTreo	
**Number of patients**	30	30	**p value**	25	25	**p value**
**Patient age (years)**			0.3			0.62
**Median (min-max)**	62.8 (51.0-69.9)	61.0 (50.0-68.0)		57.4 (50.0-67.2)	57.0 (50.0-66.0)	
**[IQR]**	[57.2-63.9]	[54.3-65.0]		[53.7-61.2]	[53.0-61.0]	
**Donor age (years)**			0.9			0.51
**Median (min-max)**	35.3 (20.6-69.1)	37.0 (19.0-65.0)		39.3 (21.2-47.3)	37.0 (19.0-58.0)	
**[IQR]**	[25.6-46.8]	[29.5-51.0]		[33.2-47.3]	[28.0-49.0]	
**Interval from D_x to HCT (months)**			0.97			0.94
**Median (min-max)**	8.1 (1.5-70.5)	7.9 (1.8-135.9)		9.6 (2.0-74.7)	7.8 (1.6-86.3)	
**[IQR]**	[4.6-17.1]	[4.9-16.2]		[5.1-17.1]	[4.8-14.0]	
**Patient sex (%)**			>0.99			0.32
**- male**	80.0	80.0		60.0	72.0	
**- female**	20.0	20.0		40.0	28.0	
**HCT-CI (%)**			0.005			0.001
**- ≤ 2**	90.5	36.7		93.8	32 .0	
**- > 2**	9.5	63.3		6.2	68.0	
**Karnofsky performance score (%)**			>0.99			>0.99
**- < 90**	35.7	32.1		36.0	36.0	
**- ≥ 90**	64.3	67.9		64.0	64.0	
**Disease of secondary origin (%)**			0.06			>0.99
**- No**	70.4	93.1		82.6	82.6	
**- Yes**	29.6	6.9		17.4	17.4	

Supplementary Table S3 continued:

	FluMel	FluTreo		BuCy	FluTreo	
			p value			p value
**Pretransplant treatment (%)**			0.79			0.52
**- untreated**	44.8	50.0		45.8	40.0	
**- treated**	55.2	50.0		54.2	60.0	
**Disease stage (%)**			0.78			>0.99
**- BM blasts <10%**	43.3	40.0		36.0	36.0	
**- BM blasts 10% - 20%**	56.7	60.0		64.0	64.0	
**Disease risk (%)**			0.13			0.46
**- (very) low**	53.3	46.4		46.7	65.2	
**- intermediate**	16.7	21.4		40.0	21.7	
**- (very) high**	30.0	32.1		13.3	13.0	
**Donor type (%)**			0.56			>0.99
**- MSD**	30.0	36.7		32.0	32.0	
**- MUD**	70.0	63.3		68.0	68.0	
**Donor-patient gender combinations (%)**			>0.99			0.41
**- F to M**	17.2	16.7		16.0	24.0	
**- other combinations**	82.8	83.3		84.0	76.0	
**Donor-patient CMV serostatus (%)**			0.82			>0.99
**- neg-neg**	33.3	40.0		19.2	22.0	
**- pos-neg**	13.3	13.3		16.7	15.0	
**- neg-pos**	26.7	16.7		16.7	15.0	
**- pos-pos**	26.7	30.0		47.5	48.0	
**Graft source (%)**			>0.99			>0.99
**- BM**	0.0	0.0		16.0	0.0	
**- PB**	100.0	100.0		84.0	100.0	

FluMel fludarabine/melphalan, *BuCy* busulfan/cyclophosphamide, FluTreo treosulfan, D_x diagnosis, *HCT* haematopoietc cell transplantation, *CI* comorbidity index, BM bone marrow, *MSD* matched sibling donor, *MUD* matched unrelated donor, *F to M* female donors for male patients, *CMV* cytomegalovirus HCT, *PB* peripheral blood

P-values for comparisons of continuous variables of FluMel or BuCy to FluTreo were calculated by paired T-test; p-values for comparisons of categorical variables were calculated by paired McNemar's test or by Pearson's Chi-squared test for 2 x >2 tables.

FIGURE LEGENDS

Fig. S1 Outcome comparison of FluTreo with FluMel or BuCy by adjusted Cox regression analysis of acute myeloid leukaemia patients. Comparison of cumulative incidence of non-relapse mortality (± 95%-CI) between **S1A** FluTreo [9% (5-13%)] and FluMel [23% (18-29%)]; $p < 0.001$ **S1B** FluTreo [9% (5-13%)] and BuCy [18% (15-22%)], $p < 0.001$; comparison of overall survival between **S1C** FluTreo [73% (66-80%)] and FluMel [56% (49-63%)], $p < 0.001$ **S1D** FluTreo [73% (66-80%)] and BuCy [61% (56-65%)], $p=0.004$; non-relapse mortality curves represent cumulative incidence estimates with relapse as competing risk, overall survival curves represent product-limit estimates; FluTreo Fludarabine/Treosulfan with a total dose of 30 g/m², FluMel Fludarabine/Melphalan with a total dose of 140 mg/m²; BuCy Busulfan with a total dose of 12.8 mg/kg and Cyclophosphamide with a total dose of 120 mg/kg (all agents given intravenously).

S1A

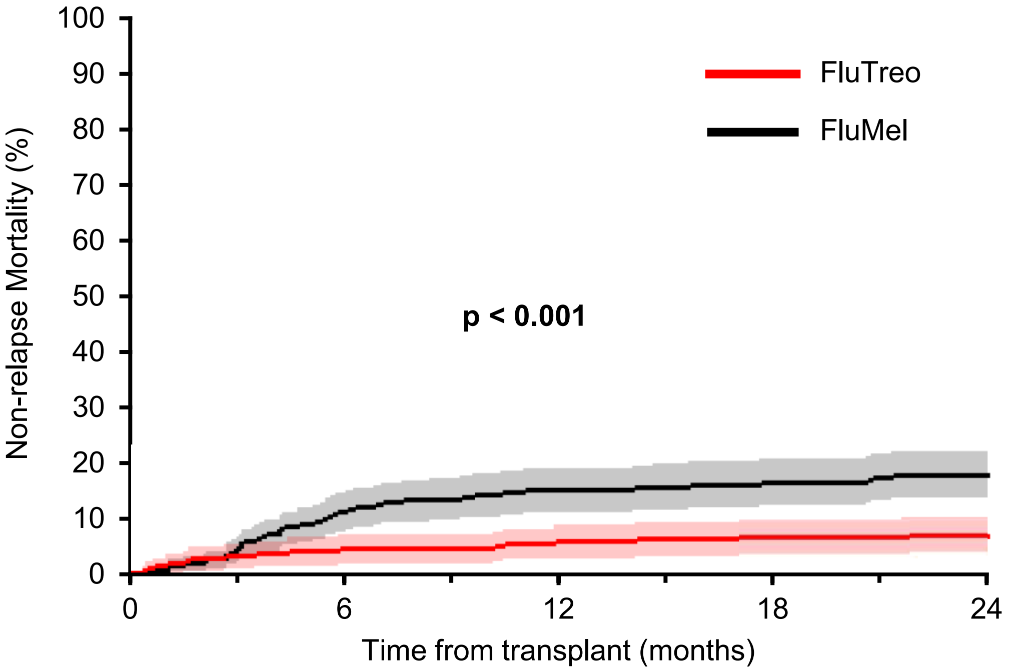

Number of at-risk patients

174	130	114	100	78
256	155	123	100	83

S1B

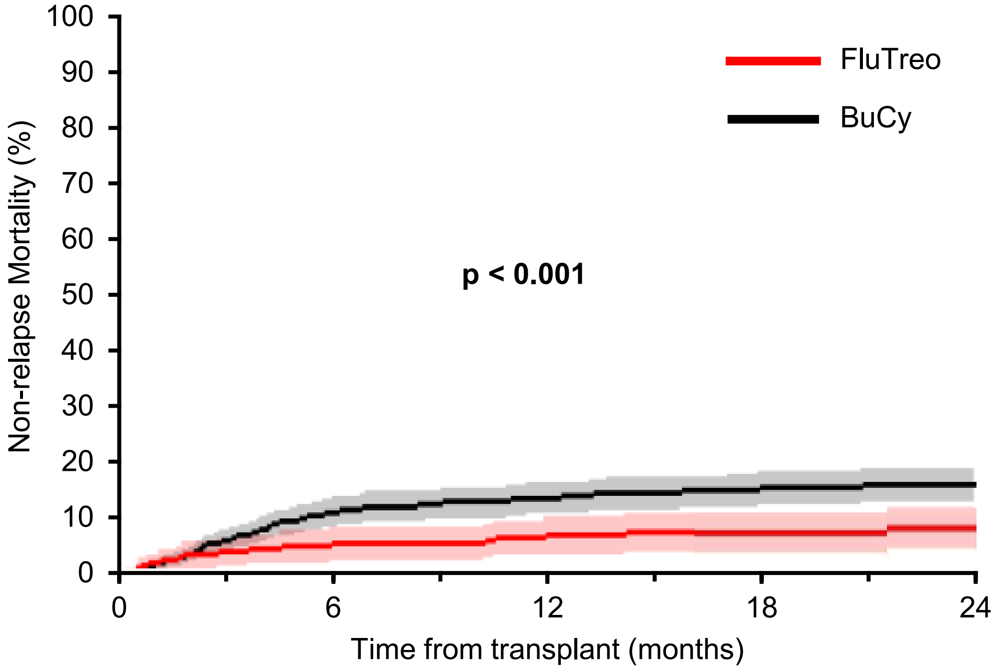

Number of at-risk patients

174	130	114	100	78
503	324	269	214	185

S1C

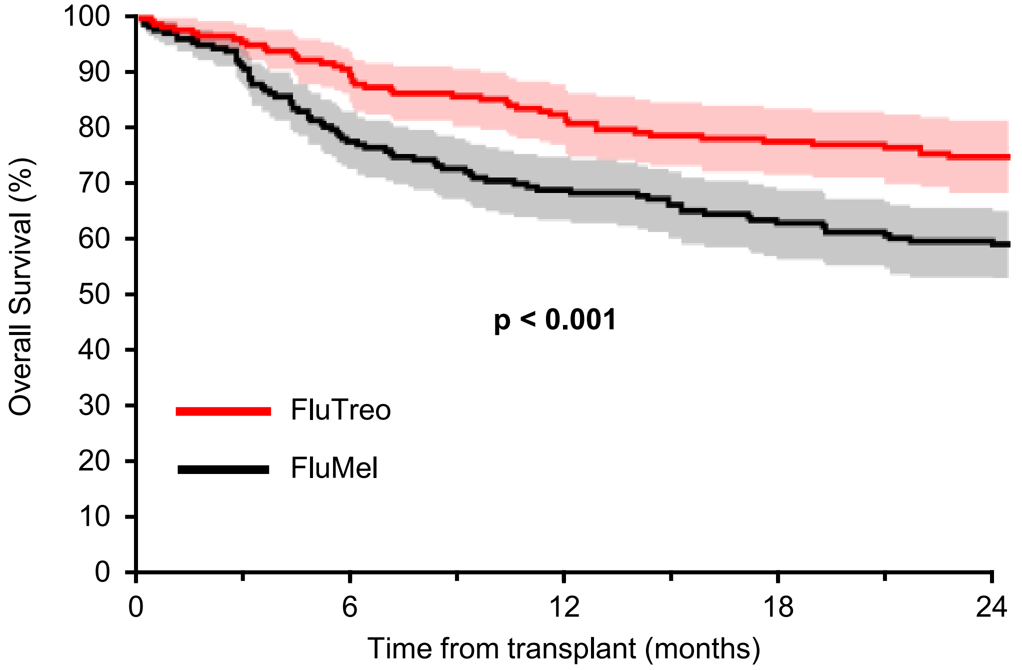

Number of at-risk patients

174	150	132	111	88
256	165	135	110	91

S1D

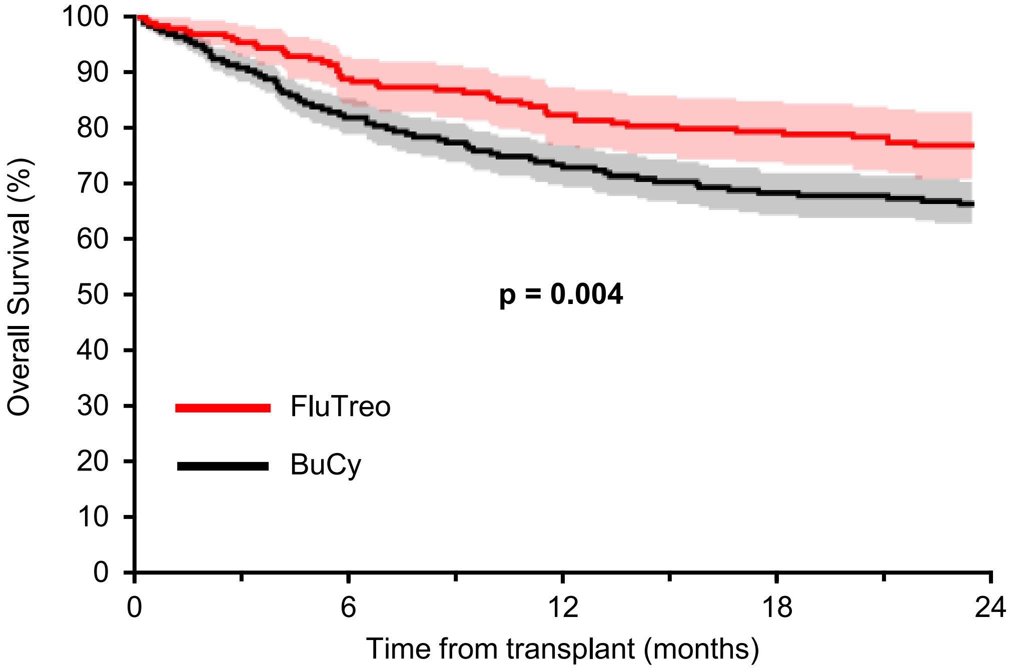

Number of at-risk patients

174	150	132	111	88
503	360	302	241	215
